# Investigating Retinal Microvascular Changes Using OCT-Angiography: The Role of Oxidative Stress and Endothelial Dysfunction in Hypertensive Nephropathy

**DOI:** 10.3390/medicina62081526

**Published:** 2026-08-08

**Authors:** Mariaelena Malvasi, Luca Salomone, Irene Azzara, Vittoria Cammisotto, Valentina Castellani, Pasquale Pignatelli, Anna Paola Mitterhofer, Silvia Lai, Francesca Tinti, Lorenzo Loffredo, Elena Pacella

**Affiliations:** 1 Department of Sense Organs, Faculty of Medicine and Dentistry, Sapienza University of Rome, 00185 Rome, Italy; 2 Nephrology Unit, Department of Translational and Precision Medicine, Sapienza University of Rome, 00185 Rome, Italy; 3 Department of Clinical Internal, Anesthesiological and Cardiovascular Sciences, Sapienza University of Rome, 00185 Rome, Italy; 4 Nephrology Unit, Department of Systems Medicine, Tor Vergata University of Rome, 00133 Rome, Italy; 5 Nephrology Unit, Department of Molecular Medicine, Sapienza University of Rome, 00185 Rome, Italy; 6 Department of Medical and Cardiovascular Sciences, Sapienza University of Rome, 00185 Rome, Italy

**Keywords:** essential hypertension, hypertensive nephropathy, optical coherence tomography (OCT), OCT angiography (OCTA), renal resistive index (RRI), oxidative stress, endothelial dysfunction, retinal microvascular damage, choroidal thickness, nitric oxide, hydrogen peroxide, microvascular disease

## Abstract

*Background and Objectives*: Arterial hypertension is a major cause of systemic microvascular damage involving target organs such as the kidneys and retina. Because the retinal and renal microcirculations share common pathogenic mechanisms, retinal imaging may provide non-invasive biomarkers of hypertensive microvascular injury. This study investigated the association between the renal resistive index (RRI), systemic oxidative stress biomarkers, and retinal abnormalities in patients with essential hypertension. Retinal structural and microvascular parameters were evaluated using optical coherence tomography (OCT) and optical coherence tomography angiography (OCTA). *Materials and Methods*: In this cross-sectional exploratory study, 32 patients with essential hypertension (64 eyes) underwent comprehensive ophthalmic examination, OCT/OCTA imaging, renal Doppler ultrasonography for RRI assessment, and evaluation of systemic oxidative stress biomarkers. Associations between renal, ocular, and biochemical parameters were analyzed. *Results*: No significant differences were observed in most global OCT/OCTA parameters. However, higher RRI values were associated with localized macular thinning and reduced optic nerve head perfusion metrics. Patients with RRI values ≥ the 75th percentile showed significantly increased hydrogen peroxide levels (*p* = 0.004) and reduced nitric oxide bioavailability (*p* = 0.033), together with thinning of selected inner macular sectors. A trend toward reduced optic nerve head flow index was also observed, suggesting early microvascular impairment that may not be detected by conventional global OCT measurements. *Conclusions*: These exploratory findings suggest a possible association among increased renal vascular resistance, oxidative stress, and localized retinal structural alterations in patients with essential hypertension. The coexistence of systemic redox imbalance and sectorial retinal changes supports the hypothesis of an eye–kidney–redox interplay and indicates that advanced retinal imaging, particularly detailed sectorial OCT analysis, may represent a complementary non-invasive tool for the early detection of subclinical microvascular damage. Larger prospective studies are warranted to validate these findings.

## 1. Introduction

Hypertensive nephropathy represents a major contributor to cardiovascular and renal morbidity and mortality, reflecting the systemic impact of chronic arterial hypertension on target organs [1,2]. At the core of this process lies microvascular dysfunction, characterized by endothelial impairment, altered vascular tone, oxidative stress, and progressive capillary rarefaction, which both precede and accompany overt organ damage [3,4,5,6,7]. In this context, the eye provides a unique and accessible window into systemic vascular health. The retinal and choroidal circulations, owing to their highly organized microvascular architecture and direct optical accessibility, allow the non-invasive, repeatable, and quantitative assessment of microvascular integrity [8,9,10,11]. Beyond vascular diseases, retinal imaging biomarkers have also shown potential utility in the early detection and monitoring of neurodegenerative and systemic disorders, further supporting the role of the retina as a surrogate marker of systemic pathology [12].

Over the past decade, advances in optical coherence tomography (OCT) and optical coherence tomography angiography (OCTA) have shifted ocular imaging from a purely morphological evaluation toward a comprehensive structural–functional characterization of retinal and choroidal microcirculation [9,13]. Quantitative OCT/OCTA parameters, including vessel density within the superficial and deep retinal capillary plexuses, foveal avascular zone (FAZ) metrics, choriocapillaris flow, retinal nerve fiber layer (RNFL), and retinal and choroidal thicknesses, have increasingly been associated with systemic vascular risk factors, endothelial dysfunction, and chronic kidney disease (CKD) progression [8,9,14,15,16,17,18]. A recent systematic review and meta-analysis demonstrated significant retinal thinning, including the retinal nerve fiber layer and ganglion cell layer, in patients with chronic kidney disease compared with healthy controls. These findings were further corroborated by a subsequent systematic review reporting consistent structural and microvascular retinal abnormalities on OCT and OCT angiography across different stages of renal disease, supporting the concept of the retina as a non-invasive biomarker of systemic vascular injury [19,20]. Collectively, these observations reinforce the potential role of retinal imaging as an early and accessible biomarker of systemic microvascular dysfunction.

In parallel, the renal resistive index (RRI), assessed by Doppler ultrasonography, has emerged as a reproducible hemodynamic marker reflecting intrarenal vascular resistance, arterial stiffness, and systemic vascular dysfunction, with established prognostic value for both renal and cardiovascular outcomes [21]. Increasing evidence suggests that an elevated RRI may reflect diffuse microvascular remodeling extending beyond the kidney and potentially involving other highly vascularized organs, including the retina [21].

Unlike conventional renal function markers, such as eGFR or albuminuria, the RRI reflects renal vascular hemodynamics and arterial stiffness, thereby providing complementary information on systemic microvascular dysfunction.

In particular, oxidative stress and endothelial dysfunction, recognized as central drivers of hypertensive microvascular damage, have rarely been investigated within a unified, multimodal framework combining OCT/OCTA metrics, RRI, and redox biomarkers [3,4,5,6,7]. Increased activation of Nicotinamide Adenine Dinucleotide Phosphate (NADPH) oxidase, particularly the Nox2 isoform, promotes reactive oxygen species (ROS) generation and reduces nitric oxide (NO) bioavailability [7], leading to endothelial dysfunction, vasoconstriction, and progressive microvascular impairment [1]. Recent evidence indicates that NOX2-derived oxidative stress and chronic low-grade inflammation are major contributors to endothelial dysfunction and systemic microvascular damage, supporting their role as pathogenic mechanisms underlying several systemic diseases [22].

However, no study has simultaneously integrated the RRI, OCT/OCTA metrics, and oxidative stress biomarkers in hypertensive nephropathy.

Emerging evidence suggests that hypertensive microvascular damage may evolve through progressive stages, beginning with oxidative stress and endothelial dysfunction, followed by localized retinal structural alterations, and ultimately leading to diffuse microvascular remodeling detectable by advanced OCTA metrics [23,24]. Within this framework, sectorial retinal analysis may provide greater sensitivity for identifying early subclinical microvascular injury before global retinal vascular impairment becomes clinically apparent [24,25].

Recent OCT and OCTA studies have demonstrated that patients with hypertension and chronic kidney disease exhibit retinal structural and microvascular abnormalities, supporting the concept of the retina as a non-invasive biomarker of systemic microvascular dysfunction. A recent systematic review and meta-analysis confirmed significant thinning of the retinal and ganglion cell layers in patients with CKD, while a subsequent systematic review further demonstrated consistent OCT and OCTA abnormalities across different stages of renal disease, reinforcing the existence of a close eye–kidney relationship [19,20].

However, most previous studies have primarily correlated retinal findings with conventional renal function markers, such as the estimated glomerular filtration rate (eGFR) or albuminuria. In contrast, the renal resistive index (RRI) reflects intrarenal vascular resistance and renal hemodynamic impairment, providing complementary information on systemic vascular stiffness and endothelial dysfunction that may precede measurable declines in renal function. Whether retinal structural and microvascular alterations are associated with an increased RRI independently of traditional renal function parameters remains poorly understood.

Accordingly, the present study was designed to investigate the interplay among renal hemodynamic impairment, retinal and choroidal structural alterations, and systemic oxidative stress in patients with essential hypertension.

Specifically, we explored the association between OCT/OCTA-derived retinal parameters and renal vascular resistance, assessed by the renal resistive index (RRI), a hemodynamic marker that provides complementary information beyond conventional renal function indices such as the estimated glomerular filtration rate (eGFR). We also evaluated the potential modulatory role of circulating oxidative stress biomarkers within this relationship. By integrating structural retinal imaging, renal hemodynamic assessment, and biochemical profiling, this multimodal approach aims to identify early, non-invasive biomarkers of systemic microvascular dysfunction and to further characterize the eye–kidney axis in hypertensive nephropathy. This integrated strategy may contribute to the development of translational vascular biomarkers for precision medicine in hypertension.

## 2. Materials and Methods

This observational, single-center study included 32 patients with essential hypertension and CKD attending the University Hospital “Policlinico Umberto I”, Sapienza University of Rome, Italy. Participants were consecutively recruited between June and November 2025. The study protocol was approved by the Ethics Committee of Sapienza University of Rome (No. 7669, Prot. 0809/2024; 11 September 2024) and was conducted in accordance with the principles of the Declaration of Helsinki. Written informed consent was obtained from all participants before enrollment.

Eligible participants were adults aged 18–70 years with a diagnosis of essential hypertension [14] and CKD stages G2–G4, defined by an eGFR between 15 and 90 mL/min/1.73 m^2^ calculated using the CKD-EPI equation according to Kidney Disease: Improving Global Outcomes (KDIGO) recommendations [15].

To ensure reliable ophthalmological assessment, only subjects with adequate visual function and fixation stability were included. Eligibility required a best-corrected visual acuity (BCVA) >4/10 (>35 ETDRS letters), expressed both in decimal notation and on the Early Treatment Diabetic Retinopathy Study (ETDRS) scale, together with the absence of anterior segment diseases, optic nerve disorders, or neuroretinal pathologies potentially affecting retinal imaging acquisition and interpretation.

Regarding exclusion criteria, patients older than 70 years, those with CKD stage G5, those undergoing renal replacement therapy, or those with a previous kidney transplantation were excluded from the study. Patients with a clinical indication for a specific dietary intervention aimed at delaying CKD progression (e.g., low-protein or very-low-protein diets or treatment with ketoanalogues), those who refused to provide informed consent, and subjects with missing data were also excluded. Furthermore, patients affected by diabetes mellitus or previous ocular disorders were not eligible for enrollment.

Ophthalmological exclusion criteria included anterior or posterior segment diseases, epiretinal membrane, significant media opacities, intravitreal anti-vascular endothelial growth factor (anti-VEGF) treatment or intraocular surgery within the previous six months, prior photodynamic therapy, spherical refractive error > 4 diopters, intraocular pressure > 18 mmHg, glaucoma, diabetic retinopathy, other chorioretinal diseases, previous ocular surgery involving either the anterior or posterior segment, and poor visual fixation. Cataract was not considered an exclusion criterion because of its high prevalence in older adults and its limited impact on retinal structure. Cataract severity was assessed during the routine ophthalmological examination using slit-lamp biomicroscopy. Eyes with media opacities resulting in inadequate OCT/OCTA image quality (signal strength < 7/10, segmentation errors, or significant motion artifacts not correctable with repeated acquisitions) were excluded from the analysis. Therefore, only scans fulfilling the predefined image quality criteria were included, minimizing the potential influence of cataract on retinal measurements.

### 2.1. Clinical and Laboratory Measurements

During the initial clinical examination, a comprehensive medical history was obtained, including comorbidities and cardiovascular and renal risk factors such as arterial hypertension, diabetes mellitus, heart failure, and metabolic syndrome. Anthropometric measurements were recorded, and routine blood tests were performed, including a complete blood count, creatinine, urea, uric acid, lipid profile, and serum electrolytes (sodium, potassium, calcium, phosphorus, and magnesium), as well as parathyroid hormone and vitamin D levels. Additionally, patients’ smoking habits and current pharmacological treatments were documented.

### 2.2. OxS Measurement

To assess oxidative stress, fasting venous blood samples were collected from all participants in the morning after an overnight fast. Blood samples were allowed to clot and were centrifuged at 300× *g* for 10 min at room temperature to obtain serum. Serum aliquots were immediately stored at −80 °C until analysis. All samples were thawed only once before analysis and were analyzed in duplicate according to the manufacturers’ instructions. Serum samples were identified by sequential numerical codes assigned at enrollment and analyzed accordingly. Laboratory personnel performing the assays were blinded to participants’ clinical status and had access only to the sample identification codes.

#### 2.2.1. Soluble NOX2-Derived Peptide (sNOX2-dp)

Serum levels of soluble NOX2-derived peptide (sNOX2-dp), a validated marker of NOX2 activation, were measured using a previously validated enzyme-linked immunosorbent assay (ELISA), performed according to the method originally developed and validated by Violi and colleagues [17]. Briefly, ELISA 96-well plates were coated overnight at 4 °C with reference standards containing known concentrations of sNOX2-dp (0–200 pg/mL) and serum samples (1 μg of total protein). After washing to remove unbound material, wells were incubated with a horseradish-peroxidase (HRP)-conjugated monoclonal antibody directed against the amino acid sequence 224–268 of the extracellular domain of NOX2. Immobilized antibody–enzyme complexes were detected by measuring HRP activity in the presence of the chromogenic substrate 3,3′,5,5′-tetramethylbenzidine (TMB), supplied with the assay reagents. The enzymatic reaction was stopped with 2 M sulfuric acid, and absorbance was measured spectrophotometrically at 450 nm. Because the increase in absorbance is directly proportional to the amount of sNOX2-dp in the sample, serum concentrations were determined by interpolation from a standard calibration curve generated in the same assay using reference standards of known concentrations. Results were expressed as pg/mL, and the intra- and inter-assay coefficients of variation were <10%. Circulating sNOX2-dp levels have been widely used as an indirect biomarker of NOX2 activation and systemic oxidative stress in both experimental and clinical studies, supporting their application in investigations of redox-related vascular dysfunction [17,26].

#### 2.2.2. Hydrogen Peroxide (H_2_O_2_)

Serum hydrogen peroxide concentrations were determined using a commercially available colorimetric detection kit (Hydrogen Peroxide Assay Kit, Abcam, Cambridge, UK; Catalog No. ab272537) following the manufacturer’s instructions. Briefly, serum samples were incubated with a chromogenic substrate and horseradish peroxidase (HRP), which catalyzes the oxidation of the substrate in the presence of H_2_O_2_ to generate a pink-colored product that is measured spectrophotometrically at 560 nm. Concentrations were calculated from a standard calibration curve and expressed in μmol/L. All samples and standards were analyzed in duplicate. The intra- and inter-assay coefficients of variation were 2.1% and 3.7%, respectively.

#### 2.2.3. Nitric Oxide Metabolites (NOx)

Due to the short half-life and intrinsic instability of nitric oxide (NO), circulating NO bioavailability was assessed by measuring its stable oxidation products, nitrate (NO_3_^−^) and nitrite (NO_2_^−^), collectively referred to as NOx [18]. Serum NOx concentrations were measured using a commercially available colorimetric assay kit (DetectX^®^ Nitric Oxide Colorimetric Detection Kit, Arbor Assays, Ann Arbor, MI, USA; Catalog No. K023-H1) according to the manufacturer’s instructions. Briefly, serum samples were diluted (1:20) and analyzed in duplicate. Total NO was determined following the enzymatic reduction of nitrate to nitrite by nitrate reductase and subsequent colorimetric detection using the Griess reaction. Concentrations were calculated from a standard calibration curve (3.125–200 μM) and expressed in μM NOx. The assay’s sensitivity was 1.02 μM, while the intra- and inter-assay coefficients of variation were 2.9% and 1.7%, respectively.

#### 2.2.4. Endothelin-1 (ET-1)

Serum endothelin-1 (ET-1) concentrations were measured using a commercially available enzyme-linked immunosorbent assay (ELISA) kit (ElabScience, Houston, TX, USA; Catalog No. E-EL-H0064) according to the manufacturer’s instructions. Briefly, serum samples and calibration standards were added to microplate wells pre-coated with a monoclonal antibody specific for human ET-1. Following incubation and washing steps, an HRP-conjugated detection antibody was added, and the immunoreaction was visualized using 3,3′,5,5′-tetramethylbenzidine (TMB) substrate. The reaction was stopped with the supplied stop solution, and absorbance was measured at 450 nm. ET-1 concentrations were determined by interpolation from a standard calibration curve ranging from 1.25 to 1000 pg/mL and expressed in pg/mL. All samples and standards were analyzed in duplicate. The intra- and inter-assay coefficients of variation were both below 10%.

### 2.3. Optical Coherence Tomography Assessment

All ophthalmic examinations, including structural OCT and OCT angiography (OCTA), were performed on the same day using the same device (Cirrus HD-OCT 6000, Carl Zeiss Meditec, Dublin, CA, USA) equipped with the proprietary AngioPlex™ software (version 11.5.2, Carl Zeiss Meditec, Dublin, CA, USA). All OCT/OCTA scans were acquired by the same ophthalmologist, who had more than 5 years of experience in retinal imaging, to ensure consistency in image acquisition. High-resolution spectral-domain imaging was obtained with the FastTrac™ eye-tracking system enabled to minimize motion artifacts. All scans were acquired with subjects seated and properly aligned, in accordance with the OSCAR-IB criteria and APOSTEL 2.0 recommendations, with fixation on the internal target [27,28]. All quantitative OCT and OCTA measurements were automatically generated by the manufacturer’s proprietary software algorithms (AngioPlex™, Carl Zeiss Meditec), without external image processing, manual binarization, user-defined thresholding, or post-acquisition modifications. Image quality and segmentation accuracy were subsequently reviewed by a senior ophthalmologist with extensive experience in retinal imaging. Only images with a signal strength ≥7/10 were included. Scans showing motion or segmentation artifacts, or inadequate image quality, were excluded and reacquired when necessary. In cases of unstable fixation or persistent motion artifacts, repeated scans were performed to obtain at least three high-quality acquisitions, and the scan with the best signal strength and segmentation quality was selected for analysis. For structural OCT, macular cube scans (512 × 128) covering a 6 × 6 mm area centered on the fovea were acquired. Automated segmentation provided macular thickness and volume (Vol, mm^3^), as well as ganglion cell complex (GCC) analysis, including foveal GCC thickness. Retinal nerve fiber layer (RNFL) thickness (µm) was assessed using the Optic Disk Cube 200 × 200 protocol, with peripapillary RNFL measurements calculated along a 3.46 mm diameter circle centered on the optic nerve head.

For OCTA, 6 × 6 mm macular scans were obtained using the AngioPlex™ platform (Carl Zeiss Meditec, Inc., Dublin, CA, USA). The superficial and deep retinal capillary plexuses were automatically segmented using the manufacturer’s predefined retinal slabs implemented in the proprietary AngioPlex™ software, version 11.5.2 (Carl Zeiss Meditec, Inc., Dublin, CA, USA). Segmentation boundaries corresponded to the default retinal layer definitions provided by the manufacturer, without manual adjustment. Segmentation accuracy was reviewed on the corresponding B-scans, and scans showing segmentation errors were excluded and reacquired when necessary. Vessel density and perfusion density were automatically calculated by the proprietary AngioPlex™ software within the ETDRS grid and corresponding sectorial subdivisions. Perfusion density was defined as the total area occupied by perfused vasculature per unit area, whereas vessel density represented the total length of perfused vasculature per unit area [29]. No external image processing, manual binarization, user-defined thresholding, or post-processing algorithms were applied. The foveal avascular zone (FAZ, mm^2^) area was automatically quantified at the level of the superficial capillary plexus. Projection artifact correction and all quantitative OCTA metrics, including the ONH flow index, were automatically generated by the proprietary AngioPlex™ software according to the manufacturer’s implementation.

Additionally, optic nerve head (ONH) angiography was performed using a 4.5 × 4.5 mm scan centered on the optic disk. The ONH flow index was automatically calculated by the proprietary software as the average decorrelation signal within the analyzed region, reflecting blood flow. Perfusion density was defined as the total area of perfused vasculature per unit area, while the flow index represented the average decorrelation signal within the analyzed region, reflecting blood flow.

**Figure 1:**
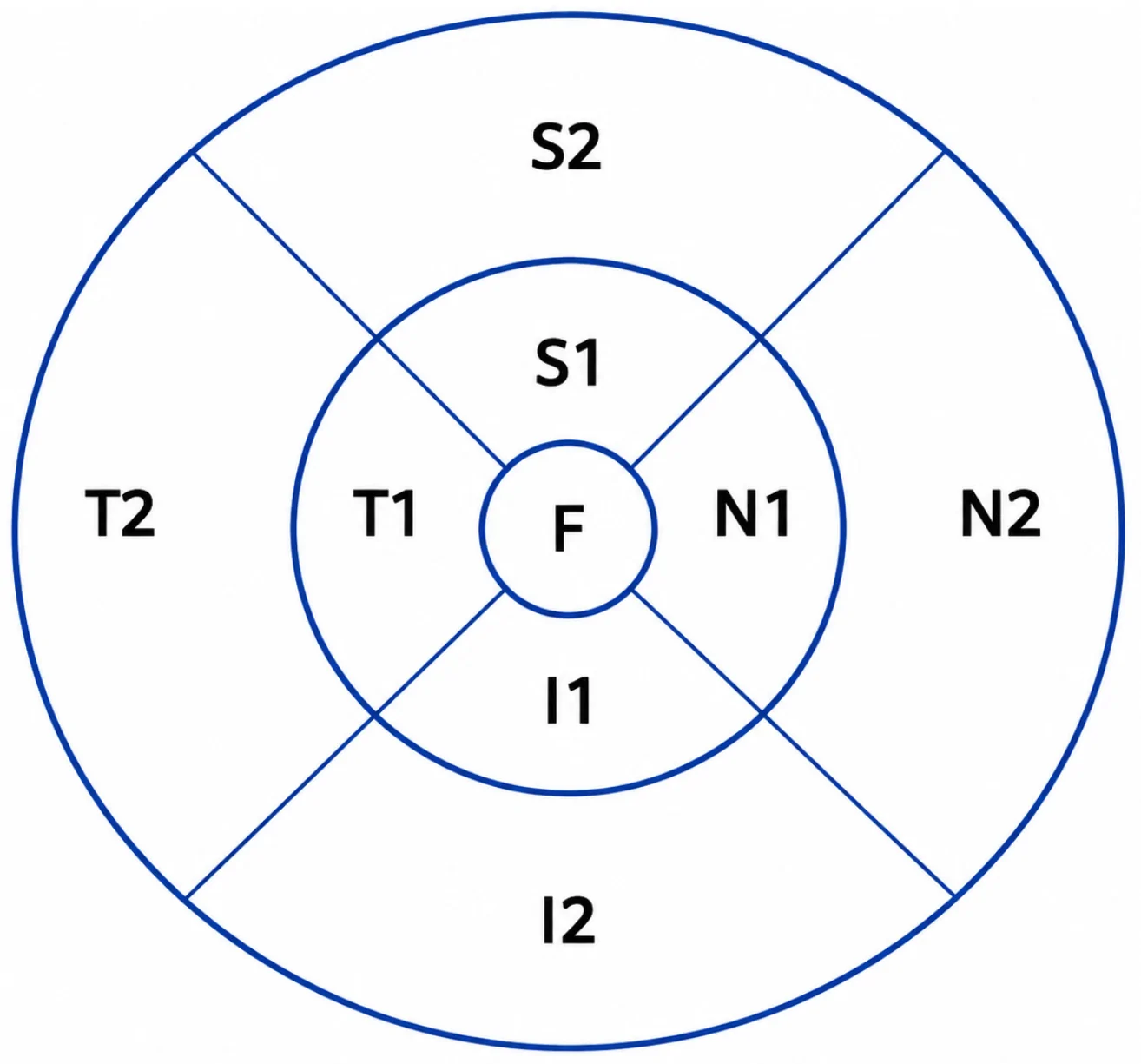
Original schematic representation of the ETDRS macular grid used for sectorial OCT and OCTA analysis. The macular region was subdivided into concentric and radial sectors, including the fovea (F), inner temporal (T1), outer temporal (T2), inner nasal (N1), outer nasal (N2), inner superior (S1), outer superior (S2), inner inferior (I1), and outer inferior (I2) sectors. This subdivision was used for the sectorial evaluation of retinal thickness and OCTA-derived vascular parameters. Adapted with permission from Ref. [12]. Copyright 2025, SAGE Publications, distributed under the terms of the Creative Commons Attribution License.

Macular thickness values were obtained for each sector and subdivided into specific subsectors to better detect localized changes, according to a radial grid reflecting the device analysis pattern, as reported in Figure 1. Measurements were recorded at 360° across the following sectors: fovea, inner temporal (Temporal 1), outer temporal (Temporal 2), inner nasal (Nasal 1), outer nasal (Nasal 2), inner superior (Superior 1), outer superior (Superior 2), inner inferior (Inferior 1), and outer inferior (Inferior 2), along with total macular volume.

The average macular thickness for each quadrant was expressed in micrometers (µm), while the volume was expressed in cubic millimeters (mm^3^). At least three measurements were obtained for each eye, and only high-quality scans with a signal strength index > 6 were retained for analysis.

The primary endpoint of the study was the assessment of the association between the renal resistive index (RRI) and OCT-derived retinal parameters, with a particular focus on sectorial macular thickness.

Secondary endpoints included the evaluation of the relationship between oxidative stress biomarkers (nitric oxide, sNOX2-dp, hydrogen peroxide) and OCT/OCTA parameters, and secondly, the assessment of the association between oxidative stress markers and renal hemodynamic impairment, as expressed by the RRI.

### 2.4. RRI Stratification

The distribution of the renal resistive index was assessed at the patient level. The 75th percentile of the RRI distribution corresponded to a value of 0.663. Therefore, participants were categorized as having lower renal vascular resistance when the RRI was <0.663 and as having higher renal vascular resistance when the RRI was ≥0.663. This cohort-derived threshold was used for exploratory stratification and was not intended to represent a universally validated pathological cutoff.

### 2.5. Statistical Analysis

Categorical variables were reported as numbers and percentages which were compared with Pearson’s χ^2^ test. Mean and standard deviation (SD) or median and interquartile range (IQR) were used for continuous variables, which were compared by Student’s *t*-test or the Mann–Whitney U test, respectively. Normal distribution of variables was checked by the Kolmogorov–Smirnov test. We used Student’s *t*-test and Pearson’s correlation analysis to evaluate normally distributed continuous variables and an appropriate nonparametric test (Mann–Whitney U test and Spearman’s rank correlation test) for the other variables. Group comparisons were performed using Fisher’s F-test (ANOVA) or the Kruskal–Wallis test when needed. Clinical and laboratory characteristics were described according to RRI percentiles. Correlations between variables were assessed via Pearson’s or Spearman’s correlation coefficients, based on data distribution. Bonferroni correction was considered for multiple comparisons, when applicable.

All tests were 2-tailed and only *p*-values < 0.05 were considered statistically significant. The analyses were performed using SPSS 25.0 software (IBM, Armonk, NY, USA).

Statistical analyses were performed at the eye level, including data from 64 eyes of 32 patients. No statistical adjustment for inter-eye correlation was applied.

This analytical approach was adopted because of the exploratory nature of the study and the limited number of participants; however, the non-independence of fellow eyes should be considered when interpreting the reported estimates and *p*-values. Future confirmatory studies with larger cohorts should employ mixed-effect models or generalized estimating equations to appropriately account for within-subject clustering. Given the exploratory nature of the study and the relatively small sample size, no multivariable regression models were used in order to avoid model overfitting. Accordingly, all statistical analyses should be considered exploratory and hypothesis-generating [30].

## 3. Results

### 3.1. Population Characteristics

The study population included 32 hypertensive patients (64 eyes), stratified according to RRI values below or above the 75th percentile. All ophthalmological analyses were conducted at the eye level.

Clinical characteristics were generally comparable between groups, with no significant differences in age, body mass index (BMI), blood pressure values, smoking status, dyslipidemia, or eGFR.

Patients with a higher RRI showed significantly lower nitric oxide levels (*p* = 0.033) and significantly higher hydrogen peroxide concentrations (*p* = 0.004), consistent with increased oxidative stress and endothelial dysfunction.

Baseline clinical, biochemical and ocular characteristics according to RRI stratification are reported in Table 1. Patients with RRI ≥75th percentile showed lower nitric oxide levels and higher hydrogen peroxide concentrations, together with localized reductions in selected macular sectors (Table 1).

The 75th percentile corresponded to an RRI value of 0.663. Accordingly, higher renal vascular resistance was defined as RRI ≥ 0.663 [31].

### 3.2. Oxidative Stress vs. RRI

Stratified analysis revealed significant differences in oxidative stress markers according to RRI levels.

Conversely, nitric oxide (NO) levels were significantly reduced in the high-RRI group (*p* = 0.033), suggesting impaired endothelial function and reduced vasodilatory capacity. No statistically significant differences were observed for sNOX2-dp levels between groups.

Patients with RRI ≥ 75th percentile exhibited significantly higher serum hydrogen peroxide concentrations (19.0 ± 6.4 µmol/L vs. 14.8 ± 4.6 µmol/L, *p* = 0.004). Conversely, nitric oxide concentrations were significantly lower in the high-RRI group (12.3 ± 8.9 µM vs. 16.9 ± 7.5 µM, *p* = 0.033), suggesting impaired endothelial function and reduced nitric oxide bioavailability. No statistically significant differences were observed for sNOX2-dp concentrations (23.4 ± 7.3 vs. 21.6 ± 8.2 pg/mL, *p* = 0.368).

These findings indicate that increased renal vascular resistance is associated with a pro-oxidative state, characterized by increased reactive oxygen species and reduced nitric oxide bioavailability. Overall, these findings indicate that higher RRI values are associated with a systemic pro-oxidative profile.

### 3.3. OCT vs. RRI

No statistically significant differences emerged for global OCT and OCTA parameters, including RNFL thickness, FAZ area, ONH perfusion, and ONH flow index.

However, sectorial macular analysis revealed localized retinal thinning in patients with RRI ≥ 75th percentile.

Specifically, exploratory analyses identified lower values in the ganglion cell complex (GCC) inner nasal sector (GCC Nasal 1) (*p* = 0.006), the GCC inner temporal sector (GCC Temporal) (*p* = 0.010), and the GCC inner superior sector (GCC Superior 1) (*p* = 0.022) compared with patients with lower RRI values, suggesting localized inner macular thinning associated with increased renal vascular stiffness.

A similar trend was observed for GCC Nasal 2 (*p* = 0.070) and ONH flow index (*p* = 0.060). Patients with a higher RRI showed a trend toward lower ONH flow index values compared with subjects with a lower RRI (0.426 ± 0.039 vs. 0.444 ± 0.033, *p* = 0.060), suggesting possible early optic nerve head microvascular impairment associated with increased renal vascular resistance.

Correlation analysis showed inverse associations between the RRI and nitric oxide levels, the ONH flow index, and selected inner macular sectors (Table 2).

**Figure 2:**
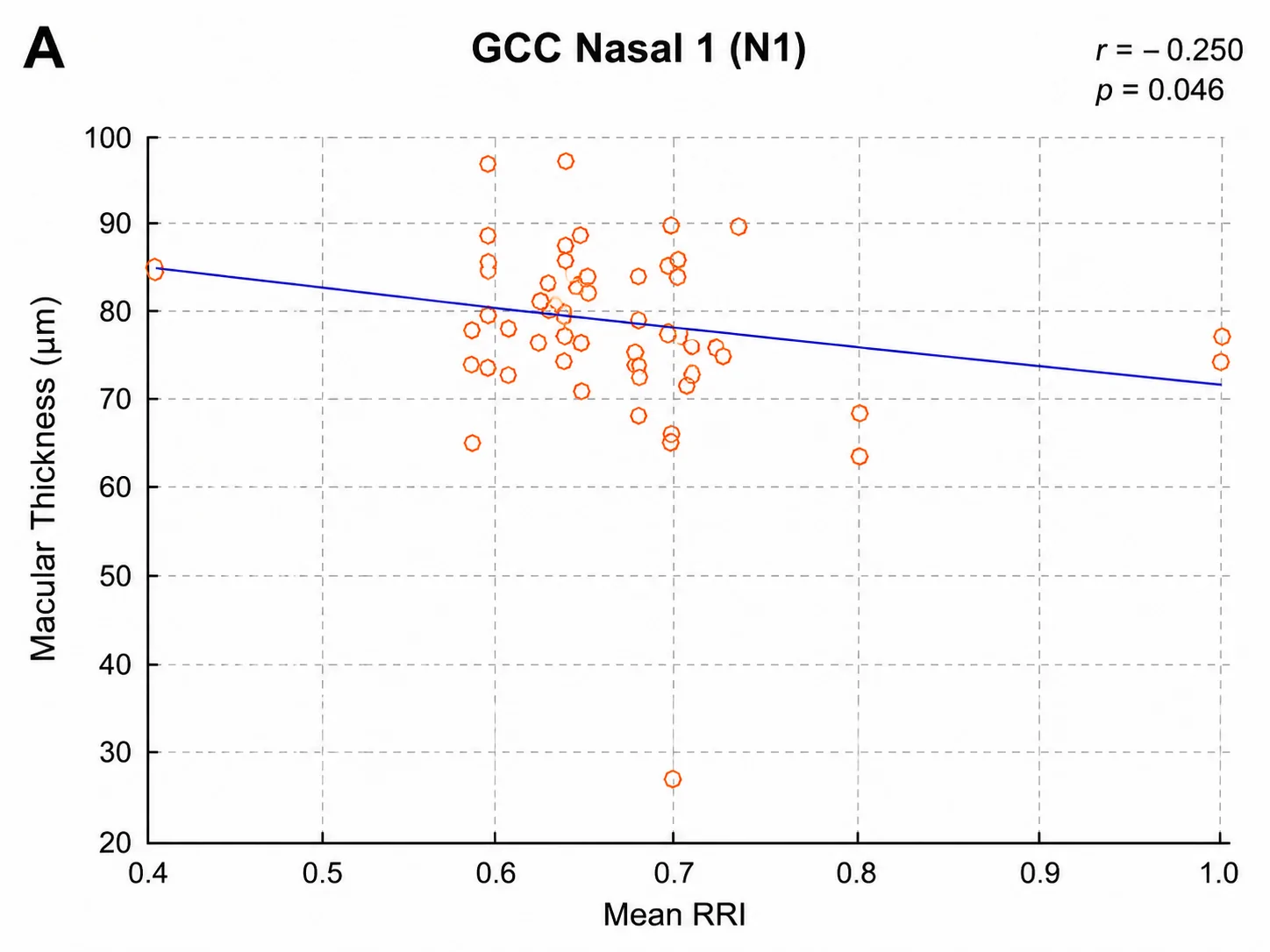

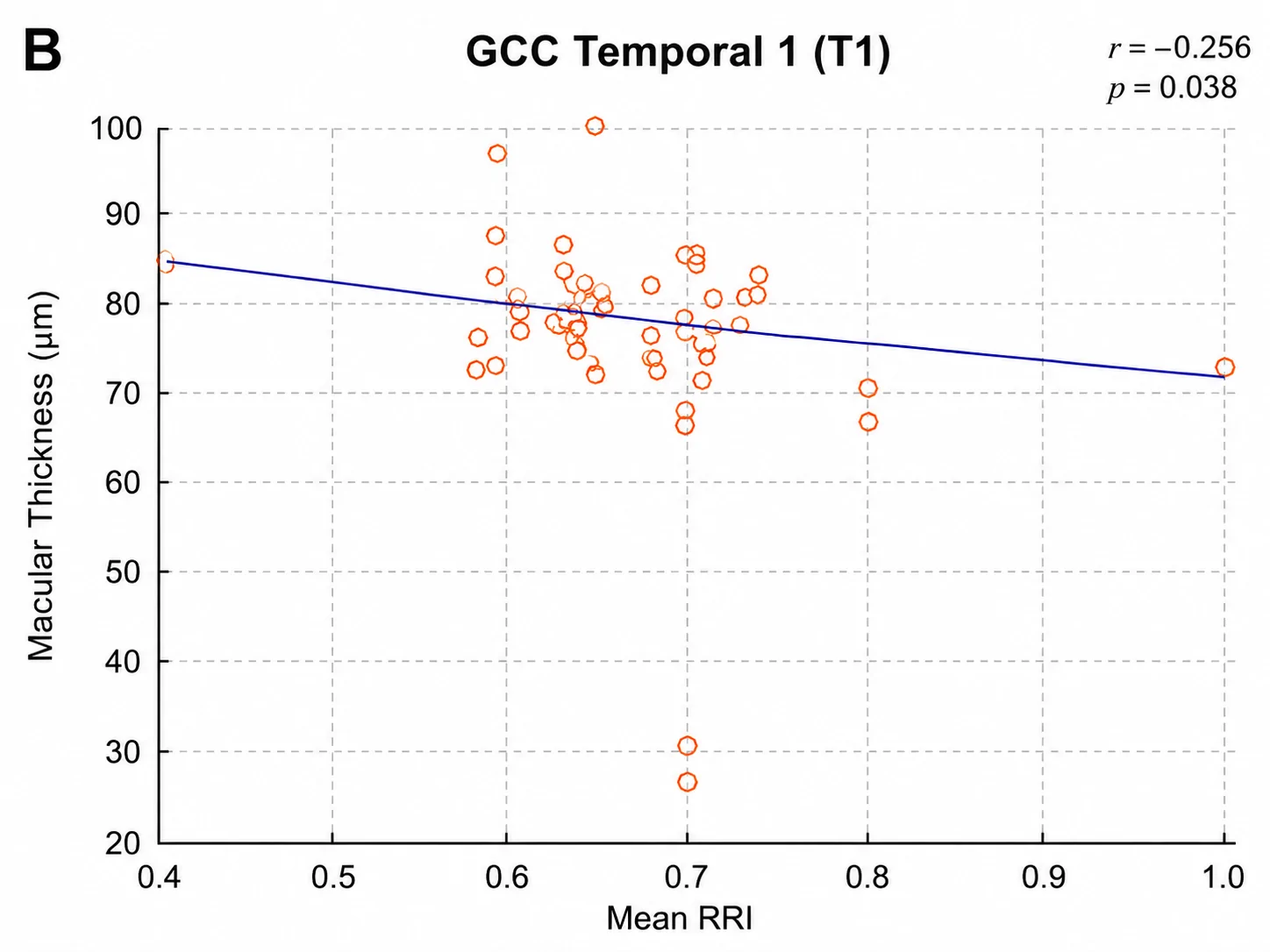

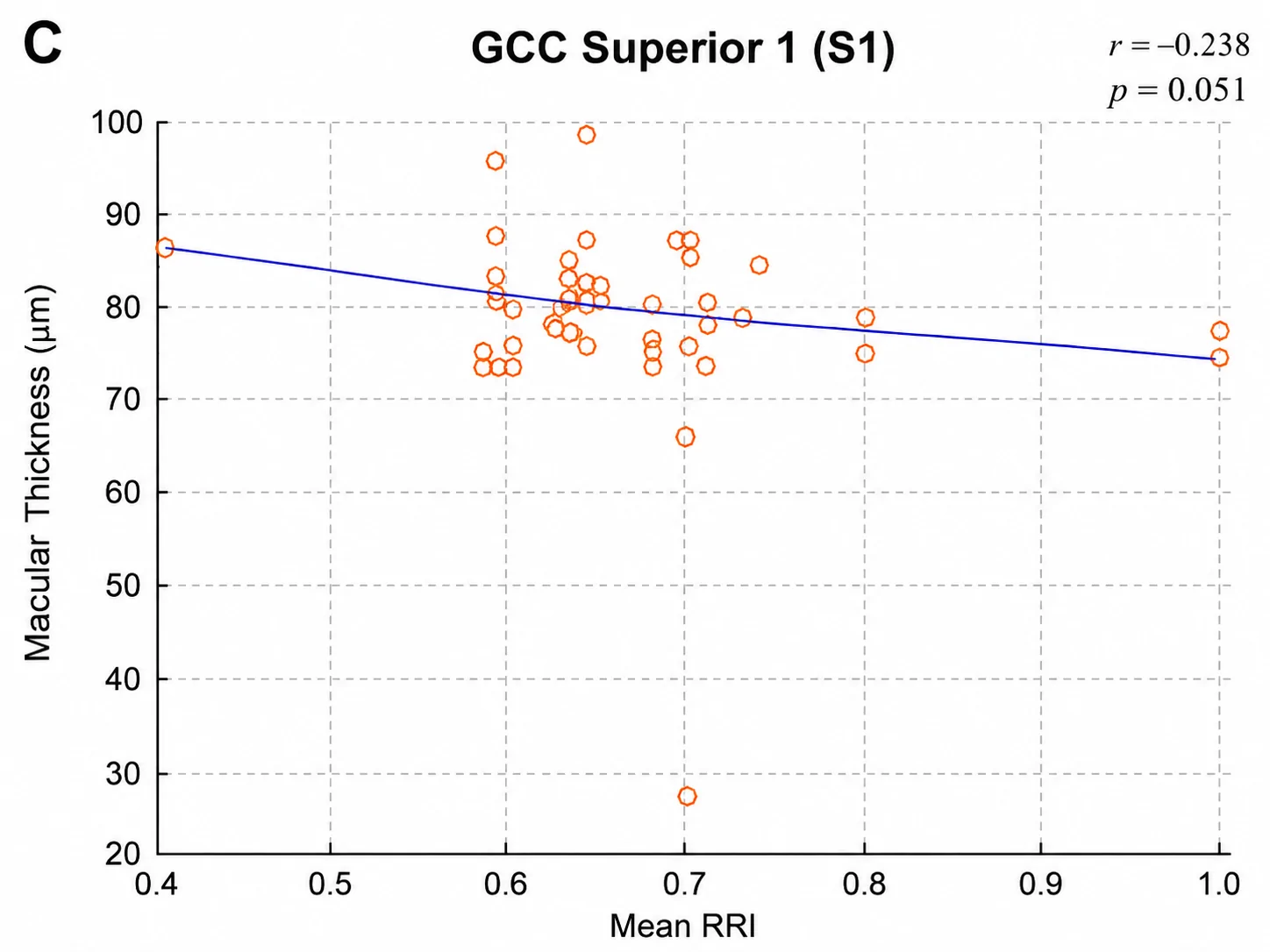
Scatter plots showing the inverse associations between renal resistive index (RRI) and macular thickness (µm) in the ganglion cell complex (GCC) Nasal 1 (r = −0.250, *p* = 0.046) (**A**), GCC Temporal 1 (r = −0.345, *p* = 0.005) (**B**), and GCC Superior 1 (r = −0.280, *p* = 0.025) (**C**) sectors. Each data point represents an individual patient included in the statistical analysis, and the solid blue line represents the linear regression fit.

Inverse correlations were observed between the RRI and GCC Nasal 1, GCC Temporal 1, and GCC Superior thickness (Figure 2).

These findings suggest that early retinal involvement in hypertensive nephropathy may preferentially affect localized inner macular sectors rather than the global retinal structure.

### 3.4. OCT vs. Oxidative Stress

Although no significant linear correlations emerged between oxidative stress biomarkers and global OCT parameters, patients with a higher oxidative stress burden exhibited the same pattern of localized inner macular thinning observed in subjects with an elevated RRI.

Patients with a more pronounced oxidative stress profile exhibited reduced thickness in selected sectorial macular regions, suggesting a possible association between systemic redox imbalance and early retinal microvascular damage.

### 3.5. OCT: Sectorial Macular Thinning

The most relevant finding of the study emerged from sectorial OCT analysis. Patients with higher renal vascular resistance (RRI ≥ 75th percentile) demonstrated localized thinning in specific inner macular sectors, namely, GCC Nasal 1 (*p* = 0.006), GCC Temporal 1 (*p* = 0.010), and GCC Superior (*p* = 0.022).

Although global OCTA parameters did not significantly differ between groups, patients with a higher RRI also showed a trend toward reduced optic nerve head (ONH) flow index values (*p* = 0.060), suggesting possible early impairment of optic nerve microvascular perfusion associated with increased renal vascular stiffness.

These changes occurred in the absence of significant differences in global OCT parameters, highlighting the presence of early and localized retinal involvement.

Importantly, this sectorial thinning pattern was associated with a pro-oxidative biochemical profile, supporting the hypothesis of a shared microvascular mechanism linking renal hemodynamic impairment and retinal structural damage.

### 3.6. OCTA Findings

No statistically significant differences were observed in OCTA-derived vascular parameters, including FAZ area, ONH perfusion, ONH flow index, and sectorial vascular density measurements.

These findings suggest that structural sectorial retinal abnormalities may precede detectable OCTA vascular changes in hypertensive microvascular disease.

**Figure 3:**
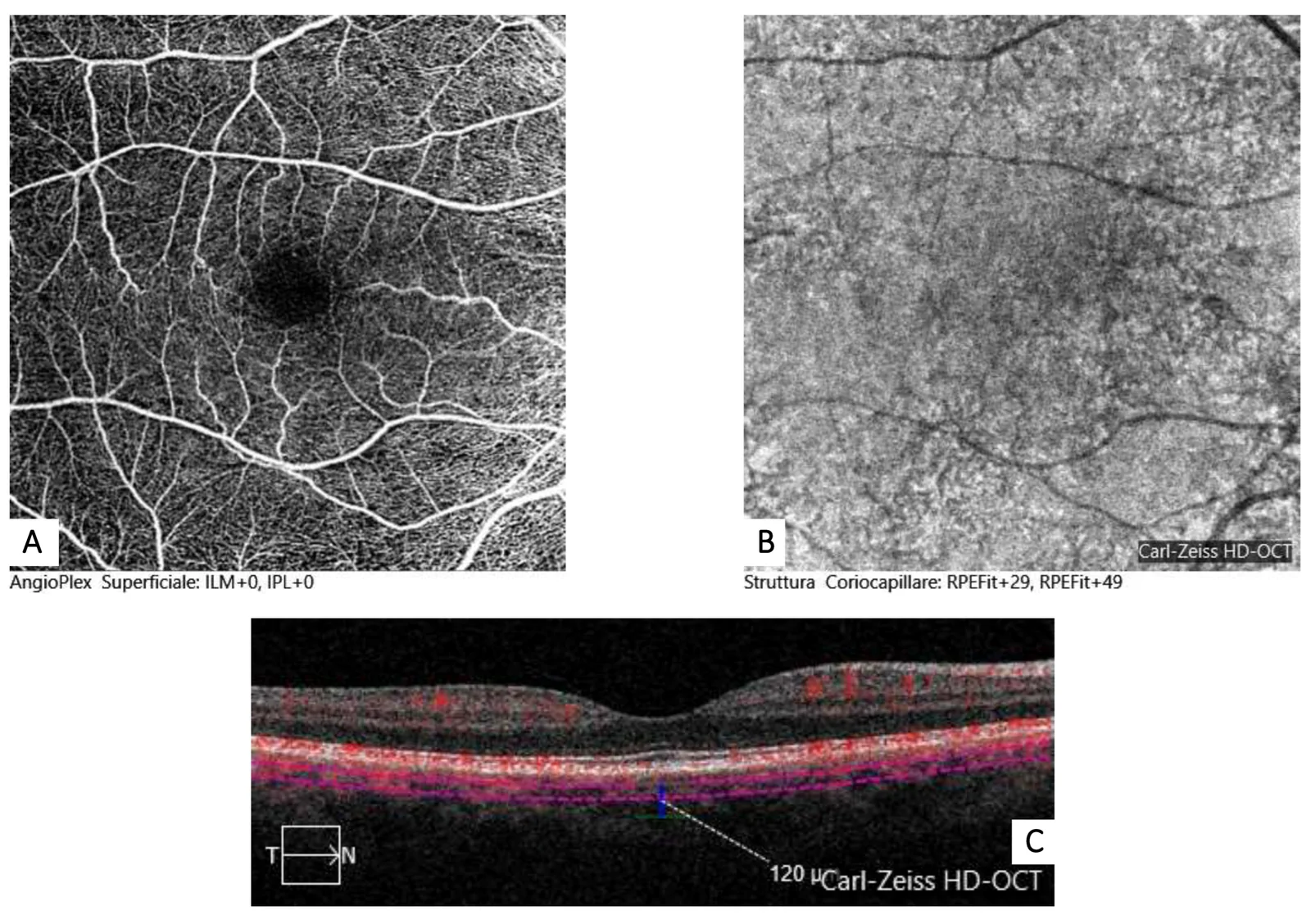
Representative multimodal OCT and OCTA findings in a patient with hypertensive nephropathy (OD). (**A**) OCT angiography of the superficial retinal capillary plexus showing mild perifoveal capillary rarefaction and reduced parafoveal vascular density. (**B**) Choriocapillaris OCTA image demonstrating heterogeneous flow distribution with focal areas of reduced signal intensity. (**C**) Structural B-scan OCT showing preserved retinal architecture and reduced subfoveal choroidal thickness (~120 μm).

**Figure 4:**
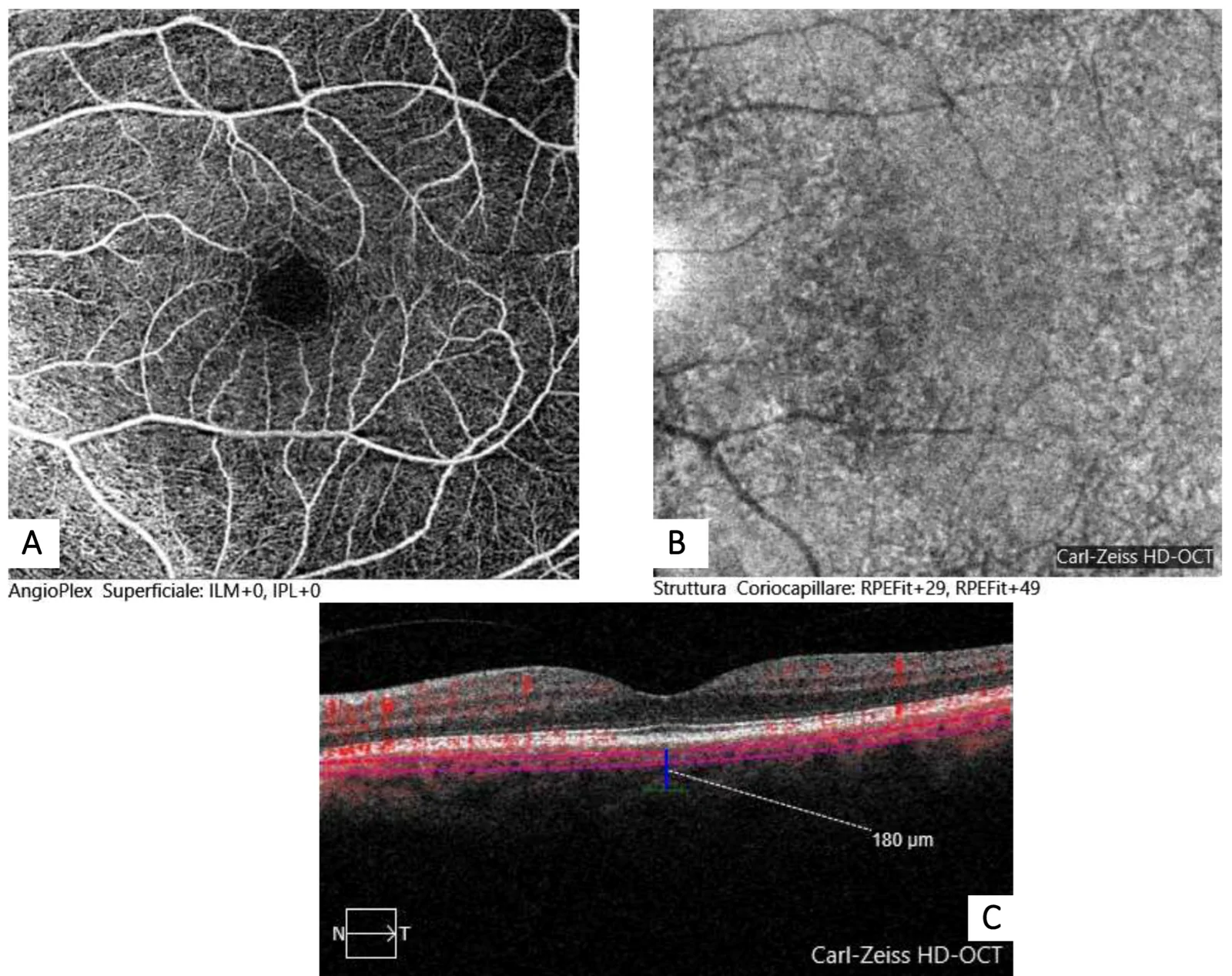
Representative multimodal OCT and OCTA findings in a patient with hypertensive nephropathy (OS). (**A**) OCT angiography of the superficial retinal capillary plexus showing mild perifoveal capillary rarefaction and reduced parafoveal vascular density. (**B**) Choriocapillaris OCTA image demonstrating heterogeneous flow distribution with focal areas of reduced signal intensity. (**C**) Structural B-scan OCT showing preserved retinal architecture and reduced subfoveal choroidal thickness (~180 μm).

Representative OCT and OCTA images illustrating retinal and choroidal microvascular alterations observed in hypertensive nephropathy are shown in Figure 3 and Figure 4.

## 4. Discussion

The present study explored the relationship among renal vascular resistance, systemic oxidative stress, and retinal structural and microvascular abnormalities in patients with hypertensive nephropathy. Although no significant linear correlations emerged between circulating oxidative stress biomarkers and global OCT/OCTA parameters, stratified analyses according to the renal resistive index (RRI) revealed a biologically coherent pattern linking oxidative imbalance with localized retinal abnormalities.

Patients with higher RRI values exhibited significantly reduced nitric oxide bioavailability together with increased hydrogen peroxide concentrations. These findings are consistent with endothelial dysfunction and an enhanced oxidative stress burden, in agreement with previous evidence demonstrating that impaired nitric oxide bioavailability is a hallmark of endothelial dysfunction in hypertension and contributes to systemic microvascular damage, including retinal microvascular alterations [32,33], and that retinal structural and microvascular alterations reflect systemic microvascular injury [24].

Notably, these biochemical alterations were associated with localized thinning of selected inner macular sectors rather than diffuse retinal structural changes. Specifically, exploratory analyses showed nominally significant reductions in the GCC Nasal 1 (*p* = 0.006), GCC Temporal 1 (*p* = 0.010), and GCC Superior sectors (*p* = 0.022) in patients with higher RRI values, supporting the presence of focal retinal involvement associated with increased renal vascular stiffness.

These findings suggest the hypothesis that hypertensive microvascular injury may initially manifest as focal retinal alterations before the development of widespread retinal remodeling [23,24,25].

Importantly, most global OCT and OCTA parameters, including RNFL thickness and FAZ area, did not differ significantly between RRI groups. Nevertheless, correlation analysis showed inverse associations between the RRI and ONH perfusion, as well as the ONH flow index, suggesting that optic nerve head microvascular parameters may be more sensitive to renal vascular resistance than other global retinal metrics. Similarly, the literature reports alterations in both macular and optic disk OCTA parameters in patients with IgA nephropathy, suggesting that optic nerve head microcirculation may also reflect systemic renal microvascular dysfunction [34].

Our findings are consistent with previous OCT and OCTA studies reporting retinal structural and microvascular abnormalities in patients with cardiovascular disease [35] and chronic kidney disease [19]. Notably, a recent systematic review confirmed that retinal thinning and OCTA abnormalities are consistently observed across different stages of renal disease, further supporting the concept of retinal imaging as a surrogate marker of systemic microvascular dysfunction [19]. Reduced retinal thickness, ganglion cell loss, and impaired retinal perfusion have also been described in patients with hypertension, coronary artery disease, heart failure, and other manifestations of systemic vascular dysfunction, reinforcing the concept that retinal tissue represents a sensitive target of systemic microvascular injury [3,8,9,10]. In this context, the association between an elevated renal resistive index (RRI) and localized macular thinning observed in our cohort extends these observations to hypertensive nephropathy. By integrating retinal imaging with renal hemodynamic assessment and circulating oxidative stress biomarkers, the present study provides a multimodal eye–kidney–redox framework, suggesting that retinal alterations may reflect a common pathophysiological pathway characterized by endothelial dysfunction, vascular stiffness, oxidative stress, and reduced nitric oxide bioavailability affecting multiple vascular beds.

This observation suggests that sectorial OCT analysis may be more sensitive than conventional global metrics in detecting early microvascular damage before overt retinal structural or vascular abnormalities become clinically evident. The recent literature supports the concept of retinal imaging as a non-invasive window into systemic vascular health [3]. OCT and OCT angiography (OCTA) have demonstrated the ability to detect systemic microvascular alterations in arterial hypertension and cardiovascular disease, even in the absence of clinically evident target-organ damage [8,9,10]. These observations reinforce the hypothesis that retinal imaging may capture subclinical vascular dysfunction before the appearance of advanced structural changes.

From a pathophysiological perspective, oxidative stress is a dynamic and time-dependent process [4,5,6,7]. Reactive oxygen species generation, endothelial responses, and vascular autoregulatory dysfunction fluctuate over time [5,6,7,13], whereas retinal structural abnormalities detectable by OCT likely reflect cumulative chronic injury. Consequently, single time-point measurements of systemic oxidative biomarkers may incompletely reflect local retinal oxidative damage [4]. Furthermore, circulating biomarkers such as NO, sNox2-DP, and H_2_O_2_ may not fully reflect the local retinal oxidative microenvironment, which is strongly influenced by blood–retinal barrier integrity, local antioxidant systems, and tissue-specific metabolic demand [5,6,13]. In line with this, previous studies have suggested that retinal injury is predominantly driven by localized oxidative stress, endothelial dysfunction, and microvascular impairment rather than systemic oxidative burden alone [5,6,7,13].

The coexistence of oxidative stress biomarkers and localized inner macular thinning supports a biologically plausible mechanism linking oxidative stress, endothelial dysfunction, and retinal microvascular injury. To our knowledge, this is among the first studies integrating renal hemodynamic assessment, systemic oxidative stress profiling, structural OCT, and OCTA-derived retinal microvascular analysis within a unified model of hypertensive microvascular dysfunction. In this framework, hydrogen peroxide may reflect enhanced oxidative damage, whereas reduced nitric oxide availability may indicate impaired endothelial-mediated vascular homeostasis and reduced vasodilatory reserve [4,5,6,7]. Experimental and clinical evidence has shown that increased NADPH oxidase activity, particularly involving the Nox2 isoform, promotes reactive oxygen species generation, nitric oxide depletion, endothelial dysfunction, and progressive microvascular rarefaction [7,28,29]. These mechanisms are closely linked to vascular aging processes characterized by endothelial senescence, impaired autoregulation, and chronic ischemic remodeling [36,37,38,39].

Our findings also support the hypothesis of a shared eye–kidney microvascular phenotype in hypertensive nephropathy [2,38,39]. Within this framework, the RRI may reflect not only intrarenal vascular resistance but also broader systemic microvascular dysfunction involving retinal circulation. Retinal regions with higher metabolic demand, particularly parafoveal inner retinal layers, may be especially vulnerable to oxidative-stress-mediated ischemic injury [5,6,7,13]. This interpretation is consistent with previous reports demonstrating retinal and choroidal thinning in patients with essential hypertension and chronic kidney disease [40,41].

Beyond structural OCT findings, OCT angiography provided additional insights into retinal microvascular alterations associated with hypertensive nephropathy. Although several global OCTA parameters did not significantly differ across the entire cohort, stratified analyses revealed a pattern of subtle localized microvascular impairment in patients with a higher RRI and oxidative stress burden. In particular, patients with an elevated RRI showed reduced vessel density in selected retinal sectors, together with a trend toward lower optic nerve head perfusion parameters. These findings suggest the hypothesis that hypertensive nephropathy is associated with early retinal microvascular dysfunction, likely reflecting systemic endothelial injury and early microvascular remodeling [2,3,8,10].

Interestingly, localized structural OCT abnormalities appeared more prominent than global OCTA alterations. Similarly, FAZ measurements remained substantially preserved across RRI strata, suggesting that diffuse retinal ischemic remodeling may not yet be established during the early phases of hypertensive microvascular disease [42]. Similar observations have been reported in retinal vascular disorders characterized by choriocapillaris dysfunction, in which focal perfusion abnormalities may precede diffuse retinal vascular remodeling detectable by conventional OCTA metrics [38,43]. Previous OCTA studies have also demonstrated localized retinal vascular alterations in systemic vascular diseases, supporting the concept of selective retinal vulnerability to microvascular dysfunction and oxidative-stress-mediated injury [36,37,38,39].

Localized retinal regions with higher metabolic demand may be more vulnerable to oxidative-stress-mediated ischemic damage, consistent with previous evidence linking focal microvascular impairment to localized retinal thinning [44]. Consequently, sectorial OCT analysis may provide greater sensitivity for detecting early hypertensive microvascular dysfunction than conventional global retinal metrics. The preservation of global OCTA parameters despite the presence of localized sectorial thinning may therefore reflect a relatively early stage of systemic microvascular disease.

These findings suggest that sectorial OCT analysis may be more sensitive than conventional global OCT and OCTA metrics for detecting early hypertensive microvascular injury, before diffuse vascular remodeling becomes clinically evident.

Taken together, these findings may support a temporal model of hypertensive microvascular injury progression. In the earliest phase, systemic oxidative stress and endothelial dysfunction may predominate, characterized by increased reactive oxygen species generation and reduced nitric oxide bioavailability [4,5,6,7,45,46]. Subsequently, localized retinal structural abnormalities, particularly sectorial inner macular thinning, may emerge as an intermediate manifestation of microvascular compromise. The selective involvement of the nasal, temporal, and superior inner macular sectors observed in our cohort is consistent with this hypothesis and may reflect regional differences in susceptibility to oxidative stress-mediated microvascular injury.

Only in later stages might diffuse OCTA abnormalities and global retinal vascular remodeling become clinically detectable [23,24,25]. Within this framework, detailed sectorial OCT analysis may represent an early and sensitive biomarker of hypertensive microvascular dysfunction [42].

Structural OCT and OCTA are likely to provide complementary information, reflecting different stages of microvascular injury [36,37,38,39,42]. While OCT identifies cumulative structural damage, OCTA may detect earlier perfusion abnormalities and microvascular remodeling.

An additional finding of potential relevance was the trend toward reduced optic nerve head flow index values in patients with a higher RRI. Although not reaching statistical significance, patients with an elevated RRI showed lower ONH flow index values compared with those with a lower RRI (0.426 ± 0.039 vs. 0.444 ± 0.033; *p* = 0.060), suggesting possible early impairment of optic nerve head microvascular perfusion associated with increased renal vascular resistance. The optic nerve head circulation is highly sensitive to vascular dysregulation and endothelial dysfunction [36,37,38,39,46]. Reduced ONH perfusion has previously been associated with systemic vascular disease, impaired vascular autoregulation, and microvascular ischemia [46]. In this context, the coexistence of increased oxidative stress, reduced nitric oxide bioavailability, and lower ONH flow may reflect an early stage of systemic microvascular compromise involving both renal and ocular circulations.

Several limitations should be acknowledged. The relatively limited sample size and cross-sectional design reduce the ability to establish causal relationships between oxidative stress, renal vascular resistance, and retinal abnormalities. Furthermore, because participants were recruited from a single tertiary referral center, a degree of selection bias cannot be excluded, and the study population may not fully represent the broader population of patients with hypertensive nephropathy. In addition, systemic oxidative stress biomarkers may not fully reflect local retinal vascular oxidative status. OCTA measurements may also be influenced by motion artifacts, segmentation variability, and signal attenuation, particularly in exploratory studies with limited sample sizes. Moreover, although all patients received standard antihypertensive treatment according to current clinical practice, the potential influence of concomitant medications on renal hemodynamics, oxidative stress biomarkers, and retinal OCT/OCTA parameters cannot be completely excluded and may have affected the observed associations. Furthermore, both eyes from each participant were included in the analysis without adjustment for inter-eye correlations. Consequently, measurements from fellow eyes cannot be considered fully independent, and this should be taken into account when interpreting the results. Given the exploratory design of the study, the inclusion of multiple retinal parameters, the relatively limited sample size, and the absence of adjustment for inter-eye correlation, the present findings should be regarded as exploratory and hypothesis-generating. In addition, the limited sample size precluded robust multivariable adjustment for potential confounding variables, including demographic and clinical characteristics; therefore, residual confounding cannot be excluded. Accordingly, the reported associations should be interpreted with caution and warrant confirmation in larger independent cohorts using statistical approaches that account for within-subject correlation and allow appropriate multivariable adjustment.

Future prospective multicenter studies, including larger and more diverse patient cohorts, are warranted to validate these findings and to better define the temporal relationship between oxidative stress, retinal microvascular alterations, and renal vascular dysfunction. Longitudinal OCT and OCTA imaging may help clarify the progression of retinal changes over time, while mechanistic studies investigating oxidative stress pathways, endothelial dysfunction, and vascular remodeling could further elucidate the biological mechanisms underlying the shared eye–kidney microvascular axis.

Overall, these findings reinforce the emerging role of multimodal retinal imaging as a non-invasive tool for investigating systemic vascular dysfunction. Detailed sectorial OCT analysis may provide greater sensitivity than conventional global retinal metrics for detecting early hypertensive microvascular injury. In addition, OCT- and OCTA-derived biomarkers may contribute to cardiovascular and renal risk stratification, supporting the concept of the retina as a surrogate marker of systemic microvascular health.

Taken together, these findings suggest the concept of the retina as a readily accessible biomarker of systemic microvascular health and reinforce the existence of a shared eye–kidney vascular axis in hypertensive nephropathy.

## 5. Conclusions

Chronic kidney disease in patients with essential hypertension appears to be associated with multisystem microvascular involvement, including the ocular circulation. In the present study, patients with higher renal resistive index values exhibited a biochemical profile consistent with increased oxidative stress, characterized by reduced nitric oxide bioavailability and increased hydrogen peroxide levels, together with localized macular thinning. These findings support the hypothesis that oxidative stress, mediated at least in part by NADPH oxidase activation and impaired nitric oxide signaling, may contribute to endothelial dysfunction and early retinal microvascular impairment [7,28].

Importantly, retinal structural abnormalities were predominantly localized and sectorial rather than diffuse, while global OCTA parameters remained relatively preserved. This observation suggests that focal retinal changes may represent an early manifestation of systemic microvascular dysfunction in hypertensive nephropathy. Within this framework, sectorial OCT analysis may provide greater sensitivity than conventional global retinal metrics for the detection of subclinical vascular injury.

Overall, multimodal retinal imaging may represent a non-invasive approach for identifying early systemic microvascular damage and investigating the eye–kidney vascular axis. OCT- and OCTA-derived biomarkers could potentially contribute to cardiovascular and renal risk stratification, with possible translational implications for personalized patient management and longitudinal monitoring of hypertensive microvascular disease.

## Figures and Tables

**Table 1:** Population characteristics. ACE-I/ARBs: ace inhibitors/angiotensin II receptor blockers, BP: blood pressure, eGFR: estimated glomerular filtration rate, OCT: optical coherence tomography. Continuous variables are presented as mean ± standard deviation (SD), and categorical variables as number (%).

Variable	Total (*n* = 64)	RRI < 75° Percentile (*n* = 42)	RRI ≥ 75° Percentile (*n* = 22)	*p*-Value
Clinical characteristics				
Age (years)	57.2 ± 14.0	56.8 ± 10.2	57.8 ± 19.6	0.787
Female (%)	46.9	42.9	54.5	0.064
Body mass index (BMI) (kg/m^2^)	24.9 ± 5.5	25.1 ± 3.4	24.4 ± 8.2	0.606
Systolic BP (mmHg)	127.5 ± 17.2	129.3 ± 12.0	124.1 ± 24.3	0.255
Diastolic BP (mmHg)	80.8 ± 8.8	80.9 ± 9.3	80.4 ± 7.7	0.854
Mean BP (mmHg)	94.2 ± 19.3	97.3 ± 9.8	88.3 ± 29.5	0.075
Smoking (%)				0.553
Former	18.8	19.0	18.2	
Active	28.1	23.8	36.4	
Dyslipidaemia (%)	34.4	28.8	45.5	0.117
Serum creatinine (mg/dL)	2.7 ± 9.5	1.0 ± 0.3	5.9 ± 15.9	0.052
eGFR (CKD-EPI) (mL/min/1.73 m^2^)	77.2 ± 19.9	74.8 ± 19.6	81.8 ± 20.3	0.184
OCT				
Nasal 1 (µm)	320.1 ± 19.1	322.9 ± 20.5	314.9 ± 15.2	0.114
Nasal 2 (µm)	279.0 ± 22.1	280.5 ± 22.6	276.1 ± 21.3	0.455
Temporal 1 (µm)	321.3 ± 17.7	323.6 ± 18.8	316.9 ± 14.8	0.153
Temporal 2 (µm)	279.5 ± 20.9	280.3 ± 20.6	277.9 ± 21.9	0.660
Superior 1 (µm)	323.9 ± 14.3	326.1 ± 13.5	319.7 ± 15.3	0.089
Superior 2 (µm)	278.0 ± 14.0	279.6 ± 12.3	275.0 ± 16.7	0.223
Inferior 1 (µm)	317.3 ± 39.3	315.9 ± 47.9	319.8 ± 12.2	0.708
Inferior 2 (µm)	306.9 ± 313.8	267.5 ± 12.1	385.9 ± 543.2	0.159
Macular Volume (mm^3^)	10.1 ± 0.4	10.1 ± 0.4	9.9 ± 0.5	0.130
Foveal GCC (µm)	65.2 ± 3.9	64.6 ±3.9	66.3 ± 4.1	0.094
GCC Nasal 1 (µm)	80.5 ± 10.4	83.0 ± 5.5	75.6 ± 15.6	0.006
GCC Nasal 2 (µm)	78.3 ± 12.1	80.3 ± 10.1	74.5 ± 14.7	0.070
GCC Temporal 1 (µm)	77.9 ± 10.3	80.3 ± 5.2	73.5 ± 15.2	0.010
GCC Temporal 2 (µm)	90.9 ± 96.4	80.5 ± 7.6	110.8 ± 164.7	0.236
GCC Superior (µm)	79.3 ± 10.7	81.5 ± 5.6	75.1 ± 15.9	0.022
GCC Inferior (µm)	76.0 ± 12.8	77.4 ± 10.5	73.5 ± 16.3	0.255
RNFL thickness (µm)	91.1 ± 8.7	91.9 ± 9.9	89.5 ± 5.5	0.305
FAZ (mm^2^)	0.3 ± 0.1	0.3 ± 0.1	0.3 ± 0.1	0.502
ONH Perfusion (%)	45.1 ± 1.7	45.3 ± 1.8	44.7 ± 1.1	0.194
ONH flow index (arbitrary units)	0.4 ± 0.04	0.4 ± 0.03	0.4 ± 0.04	0.060
Superior 1	17.9 ± 2.8	18.0 ± 2.6	17.9 ± 3.1	0.848
Superior 2	18.4 ± 2.1	18.3 ± 2.1	18.5 ± 2.1	0.855
Inferior 1	18.3 ± 1.7	18.4 ± 1.3	18.1 ± 2.3	0.612
Inferior 2	18.4 ± 1.7	18.5 ± 1.4	18.2 ± 2.3	0.566
Oxidative stress biomarkers				
Nitric Oxide (µM/L)	15.3 ± 8.3	16.9 ± 7.5	12.3 ± 8.9	0.033
sNox-2-dp (pg/mL)	22.2 ± 7.9	21.6 ± 8.2	23.4 ± 7.3	0.368
Hydrogen peroxide (μmol/L)	16.1 ± 5.6	14.8 ± 4.6	19.0 ± 6.4	0.004
Therapy				
ACE-I/ARBs (%)	78.1	81.0	72.7	0.450
Diuretics (%)	37.5	28.6	54.5	0.041
Calcium channel blockers (%)	50.0	57.1	36.4	0.114
Beta blockers (%)	43.8	42.9	45.5	0.842
Alpha blockers (%)	6.3	4.8	9.1	0.497

**Table 2:** Exploratory Spearman correlations between renal resistive index (RRI), oxidative stress biomarkers, and selected OCT/OCTA parameters.

	r	*p*-Value
Nitric oxide	−0.357	0.004
Hydrogen peroxide	0.11	0.394
sNOX2-dp	−0.019	0.881
GCC Nasal 1	−0.250	0.046
GCC Temporal 1	−0.345	0.005
GCC Superior	−0.280	0.025
RNFL thickness	−0.161	0.205
FAZ	0.219	0.159
ONH perfusion	−0.274	0.029
ONH flow index	−0.366	0.003

## Data Availability

The data presented in this study are not publicly available due to privacy and ethical restrictions.

## Abbreviations

The following abbreviations are used in this manuscript:

OCT	Optical coherence tomography
OCTA	Optical coherence tomography angiography
ONH	Optic nerve head
RNFL	Retinal nerve fiber layer
CKD	Chronic kidney disease
FAZ	Foveal avascular zone
NADPH	Nicotinamide adenine dinucleotide phosphate
ROS	Reactive oxygen species
NO	Nitric oxide
NOX2	NADPH oxidase 2
eGFR	Estimated glomerular filtration rate
KDIGO	Kidney Disease: Improving Global Outcomes
ETDRS	Early Treatment Diabetic Retinopathy Study
BCVA	Best-corrected visual acuity
VEGF	Vascular endothelial growth factor
H_2_O_2_	Hydrogen peroxide
GCC	Ganglion cell complex
SD	Standard deviation
IQR	Interquartile range
BMI	Body mass index
